# The Effects of Different Anode Positions on the Electrical Properties of Square-Silicon Drift Detector

**DOI:** 10.3390/mi13091496

**Published:** 2022-09-08

**Authors:** Wei Luo, Longjie Wang, Rui Jia, Ke Tao, Bolong Wang, Xiaoping Ouyang, Xing Li

**Affiliations:** 1 Institute of Microelectronics of the Chinese Academy of Sciences, Beijing 100029, China; luowei2020@ime.ac.cn (W.L.); wanglongjie@ime.ac.cn (L.W.); taoke@ime.ac.cn (K.T.); wangbolong@ime.ac.cn (B.W.); 2 University of Chinese Academy of Sciences, Beijing 101408, China; 3 State Key Laboratory of Intense Pulsed Radiation Simulation and Effect, Northwest Institute of Nuclear Technology, Xi’an 710024, China; oyxp2003@aliyun.com

**Keywords:** Silicon Drift Detector, anode position, device performance, energy resolution

## Abstract

The Silicon Drift Detector (SDD) with square structure is often used in pixel-type SDD arrays to reduce the dead region considerably and to improve the detector performance significantly. Usually, the anode is located in the center of the active region of the SDD with square structure (square-SDD), but the different anode positions in the square-SDD active area are also allowed. In order to explore the effect on device performance when the anode is located at different positions in the square-SDD active region, we designed two different types of square-SDD in this work, where the anode is located either in the center (SDD-1) or at the edge (SDD-2) of its active region. The simulation results of current density and potential distribution show that SDD-1 and SDD-2 have both formed a good electron drift path to make the anode collect electrons. The experimental results of device performance at the temperature range from −60 °C to 60 °C show that the anode current of the two fabricated SDDs both decreased with the decrease of temperature, but their voltage divider characteristics exhibited high stability resistance value and low temperature coefficient, thereby indicating that they could both provide corresponding continuous and uniform electric field at different temperatures. Finally, SDD-1 and SDD-2 have energy resolutions of 248 and 257 eV corresponding to the 5.9 keV photon peak of the Fe-55 radioactive source, respectively. Our experimental results demonstrate that there is no significant impact on the device performance irrespective of the anode positions in the square-SDD devices.

## 1. Introduction

Silicon Drift Detector (SDD), as an important radiation detector, was first proposed by Emilio Gatti and Pavel Rehak in 1983 [1,2]. A critical feature of SDD is the low output capacitance, which is independent on the active area of the detector. It enables SDD to have lower electronic noise, higher count rate, and better energy resolution at short shaping times when they are used in some high energy nuclear radiation physics experiments [3,4,5]. Thanks to the researchers’ continuous development of SDD in the past 40 years, they are now widely used in space exploration, X-ray spectroscopy, particle physics [6,7], etc. [8,9,10,11,12].

The traditional SDD generally adopts concentric circular ring structure or spiral structure in which the anode is located in the center of the active region since this symmetrical structure enables the signal electrons generated by radiation ionization to be efficiently transported from the periphery to the central anode along the drift path in the active region of SDD. However, the traditional SDD with concentric circular ring structure or spiral ring structure will form a too large dead region in the fabrication of pixel-type SDD arrays, resulting in inhomogeneity of signal electrons generated by ionizing radiation throughout the entire chip. To solve this problem, the SDD with square structure (square-SDD) is often used in pixel-type SDD arrays to reduce the dead region considerably and to improve the detector performance significantly [13,14,15,16]. Even though the anode is usually located in the center of square-SDD active region, the different anode positions in the square-SDD active area are also allowed, but few research and development studies have focused on this issue.

In this work, to explore the effect on device performance when the anode is located at different positions in the square-SDD active region, we designed two types of square-SDD with different anode positions, where the anode is located either in the center (SDD-1) or at the edge (SDD-2) of its active region. Firstly, the current density and potential distribution of signal electrons in the two square-SDDs were investigated by simulation. Then, we performed a comparative analysis of the device performance of the two fabricated square-SDDs at the temperature range from −60 °C to 60 °C. Along with that, the influence of temperature on anode current and voltage dividers was investigated simultaneously. Finally, the energy resolution of the two fabricated square-SDDs was measured with the Fe-55 radioactive source.

## 2. Experiment

**Figure 1:**
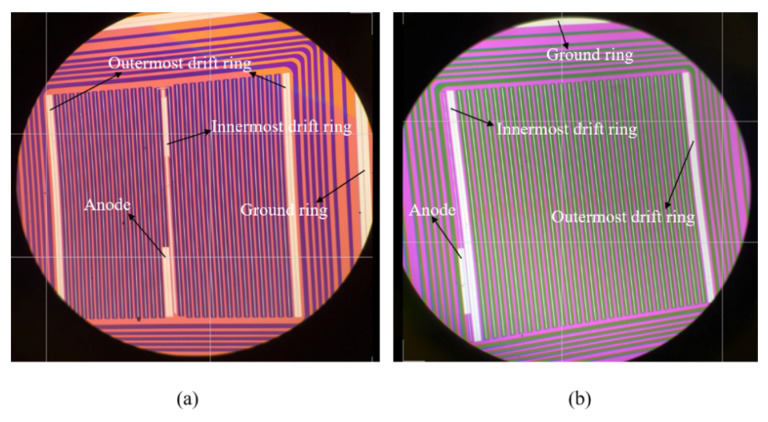
(**a**,**b**) The optical image of the fabricated two square-SDD devices SDD-1 and SDD-2. Metallic aluminum covers the surfaces of the anode, the innermost drift ring, the outermost drift ring, and the ground ring, respectively, for the applied voltage.

For the fabrication of SDD, 525 μm thick, 4-inch double-polished <100> n-type monocrystalline silicon wafers with high resistivity (>10 kΩ•cm) were used as substrates. The concentration of dopant in the high resistance silicon substrate is about 4 × 10^11^ cm^−3^. After the wafers with a standard RCA cleaning process, a 500 nm thick SiO_2_ layer was prepared on both sides of silicon substrate for photolithography and etching process to form the heavily doped N+ and P+ regions of SDD. Then, the thin phosphorus-doped and boron-doped amorphous silicon films were deposited by APCVD on those N+ regions and P+ regions, respectively. Next, a subsequent annealing process at 800 °C was used to activate the implanted boron and phosphorus atoms in amorphous silicon films. Finally, the Al electrodes were deposited on the corresponding N+ and P+ regions of SDD by electron beam evaporation and metal lift-off process in acetone was performed to form the patterned metal contacts. Some detailed fabrication processes can be seen in these publications [17,18,19,20]. We fabricated the two square-SDD devices SDD-1 and SDD-2 by APCVD technology and their structures are shown in Figure 1a,b. It can be seen that inside the active region of SDD are strip-shaped drift rings and anode. The area of active region is 9 mm^2^, the length of micro-strips is 2.8 mm, the widths of micro-strip drift rings and anode are 0.075 and 0.025 mm, respectively. The metallic aluminum covers the surfaces of these structures for the applied voltage.

For the testing of SDD electrical properties, the Semiconductor Device Parameter Analyzer (model 4200-SCS by Keithley) has been used to measure the leakage current of SDD device at different temperatures. For the measurement of energy resolution of SDD device, we have developed a special setup, and Fe-55 was used as a radioactive source. The SDD device and the ASIC (a monolithic CMOS charge sensitive preamplifier designed by the team from Tsinghua university [21,22,23]) were mounted on the specialized circuit board, and the output electrode of SDD device was bonded to the input pad of ASIC. Then, the whole system was placed in a stainless-steel vacuum chamber and cooled in liquid nitrogen. In particular, the position of the Fe-55 radioactive source was located approximately 20 mm from the SDD device inside the vacuum chamber. After that, the output of ASIC was processed by an amplifier (model 855 by Ortec). Finally, the energy resolution of SDD device can be obtained by acquiring the energy spectrum with a multi-channel analyzer (model 927 Aspec by Ortec) connected to the amplifier.

## 3. Results and Discussion

**Figure 2:**
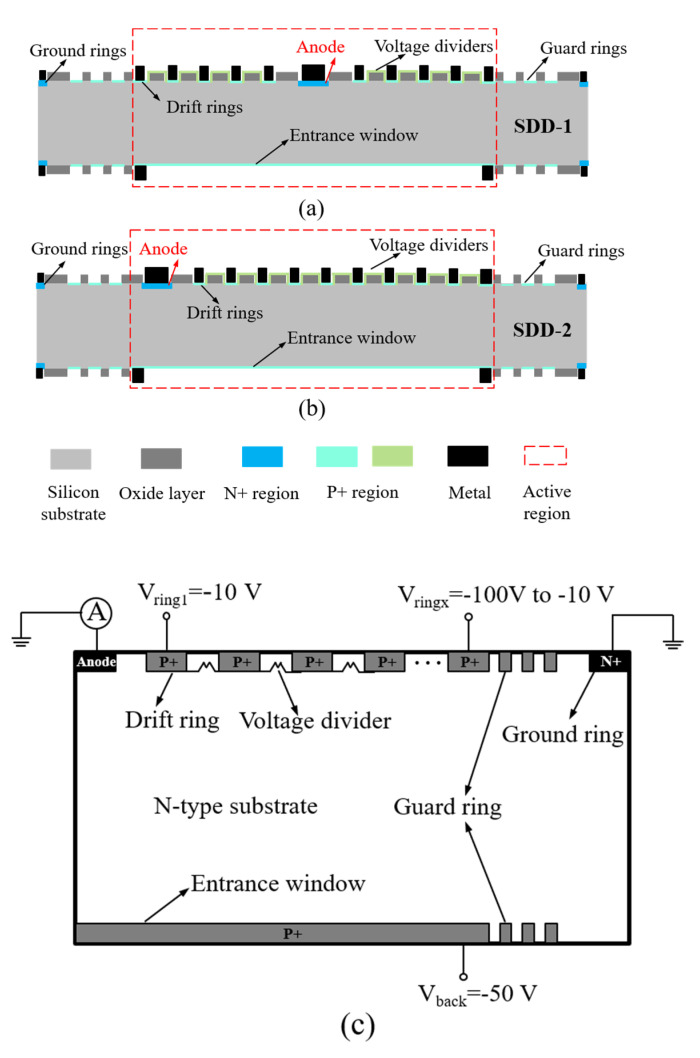
The schematic diagram of cross-section of the two square-SDD structures: (**a**) The anode is located in the center of the active region (SDD-1). (**b**) The anode is located on the left edge of the active region (SDD-2). (**c**) A schematic of the setup for measuring the anode current of the two SDDs.

To explore the effect on device performance when the anode is located at different positions in the SDD active region, we designed two different types of SDD with square structure (square-SDD) in this experiment: The anode is in the center of the active region, named SDD-1 and the anode is in the left edge of the active region, named SDD-2. The schematic diagrams of their cross-sections of them are shown in Figure 2a,b, respectively. As clearly shown in the figures of the basic device structures, one can differentiate between the two parts: Active region and non-active region according to different functions. In the active region, one side of the silicon substrate has drift rings and voltage dividers for providing a continuous and uniform lateral drift electric field. The entrance window, which is located on the opposite side of the silicon substrate, is usually used to receive the signal electrons generated by the ionization of the radiation source and offer a vertical electric field to transport them to the middle of the substrate. In the non-active region, there are guard rings and ground rings on both sides of silicon substrate, which are utilized to prevent the breakdown phenomenon occurring at the edge of the active region and to shield the leakage current generated outside the device, respectively. Figure 2c depicts a schematic of measuring the anode current of the two square-SDDs, we sweep the voltage of the outermost drift ring (ringx) from −100 to −10 V and fix the voltage of the innermost drift ring (ring1) and entrance window (back) to −10 and −50 V, respectively. Meanwhile, ensure guard rings are floating and ground rings and anode are at 0 V. Finally, under the lateral and vertical electric fields offered by the corresponding electrodes of SDD, the signal electrons will follow a specific drift path moving toward the anode and will be collected.

**Figure 3:**
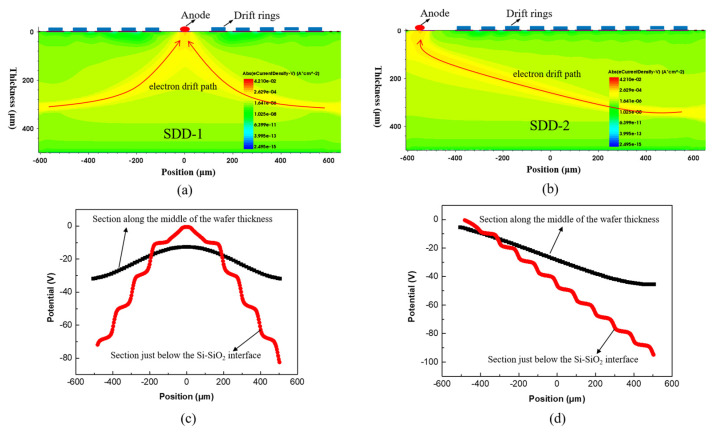
(**a**,**b**) The simulated profile of the electron current density of SDD-1 and SDD-2, respectively. The simulated structure with only the active region and the entrance window is not shown here. (**c**,**d**) The simulation of the potential distribution of SDD-1 and SDD-2 with only active region, respectively. The red line represents the potential profile below the Si-SiO_2_ interface and the black line represents the potential profile along the middle of the wafer thickness.

Before fabricating the two square-SDD devices SDD-1 and SDD-2, the current density sand potential distribution of electrons in these devices were simulated by the technology computer-aided design (TCAD) tool [24], which can help us qualitatively analyze the feasibility of device structure design. As depicted in Figure 3a,b, there are ten doped P+ drift ring regions and doped N+ anode region in the active regions of SDD-1 and SDD-2, respectively. The P+ and N+ regions are defined by gauss distribution with a surface doping concentration of 4 × 10^19^ cm^−3^ and the depth of junction of 0.2 μm. We distributed the voltages from −90 to −10 V equally spaced to the symmetric drift rings of SDD-1 and the voltages of −100 to −10 V to the equally spaced drift rings of SDD-2. By giving the same voltage of −50 V to the entrance windows (not shown in Figure 3) of SDD-1 and SDD-2 respectively, the X-ray generated electron-hole pairs will be rapidly separated under the action of the lateral drift electric field and the vertical electric field in the active region. As shown in Figure 3a, the electron current density distribution of SDD-1 indicates that the higher electron density is formed on the electron drift paths on both sides of the anode, which makes all electrons in the active region eventually follow the electron drift paths to the anode to be collected. The profile of electron current density of SDD-2 is depicted in Figure 3b. We can see that the electron density distribution trend on the electron drift path extending from right to left to the anode is similar to that of SDD-1. The two SDDs form a good electron drift path, indicating that reasonable changes in anode position within the active region of SDD do not affect the collection of electrons by the anode.

**Figure 4:**
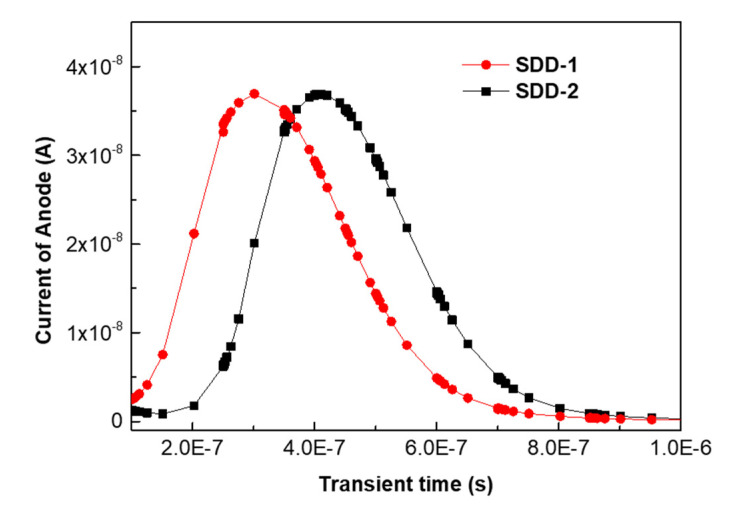
The simulation of the anode current of SDD-1 and SDD-2 versus the time for collecting electrons. In the simulation, E (the energy of incoming radiation) is 5 Mev and t (the time of collecting electrons) is 0 to 1 × 10^−6^ s.

The potential profile below the Si-SiO_2_ interface and along the middle of the wafer thickness in the active regions of SDD-1 and SDD-2 were also simulated. As shown in Figure 3c,d, for the potential distribution below the Si-SiO_2_ interface, we can find that the highest potential of the active regions of both SDDs is located at the anode, indicating that the electrons eventually flow to the anode. Similarly, the potential highest points in the middle of the wafer thickness of the two SDDs are below the anode, indicating that the flow of electrons in the middle of the wafer thickness is also towards the anode. This can explain the difference in the electron drift paths of the active regions of SDD-1 and SDD-2. In addition, we simulated the delay in the time for anode collecting electrons as the electronic drift paths of SDD-1 and SDD-2 are different. In our simulations, we set E = 5 Mev (the energy of the incoming radiation) and t = 0 to 1 × 10^−6^ s (the time of collecting electrons). As shown in Figure 4, we can find that both devices SDD-1 and SDD-2 can collect electrons in a very short time (approximately 0.4–0.6 us), and SDD-1 has a better timing performance than SDD-2. These results suggest that the different anode positions in the square SDD active region have an impact on the collection of electrons by the anode, and the anode positions that can form shorter electron drift paths are easier to collect electrons in a shorter time. Overall, we demonstrated the feasibility of both SDDs designs through simulation results.

**Figure 5:**
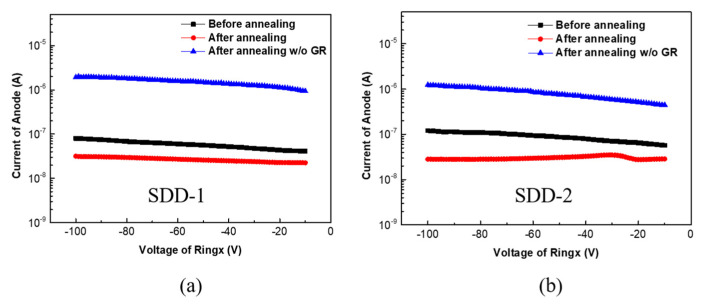
(**a**,**b**) The anode current of SDD-1 and SDD-2 at different test conditions (before annealing, after annealing and after annealing the ground ring not grounded), respectively.

According to the test method of anode current of SDD described in Figure 2c, we first investigated the device performance of SDD-1 and SDD-2 before and after annealing at room temperature. As shown in Figure 5a,b, the anode current of SDD-1 and SDD-2 before annealing are 80.2 and 121.2 nA (@−100 V), respectively. After the two samples annealing in a quartz furnace in forming gas (5% H_2_ diluted in N_2_) ambient at 400 °C for 10 min, their anode currents are decreased to 31.7 and 28.2 nA (@−100 V), respectively. It is indicated that the annealing process can help to lower the reverse current of the SDD device. This can be explained by the annealing process reducing the interface state density and forming a good ohmic contact in the Al-silicon interface, which finally reduces the leakage current of the device [25,26]. In addition, the anode current of SDD-1 and SDD-2 without ground ring grounded (*w*/*o* GR) after annealing are also performed. As depicted in Figure 5a,b, the anode current of SDD-1 and SDD-2 after annealing *w*/*o* GR is two times of magnitude higher than that of the device with ground ring grounded, indicating that the leakage current of edge termination of SDD (origin from the large number of defects generated when SDD is diced from the wafer) can be shielded when the ground ring of SDD is grounded [27]. Therefore, for SDD, better device performance can be obtained by using an annealing process and grounding the ground ring.

**Figure 6:**
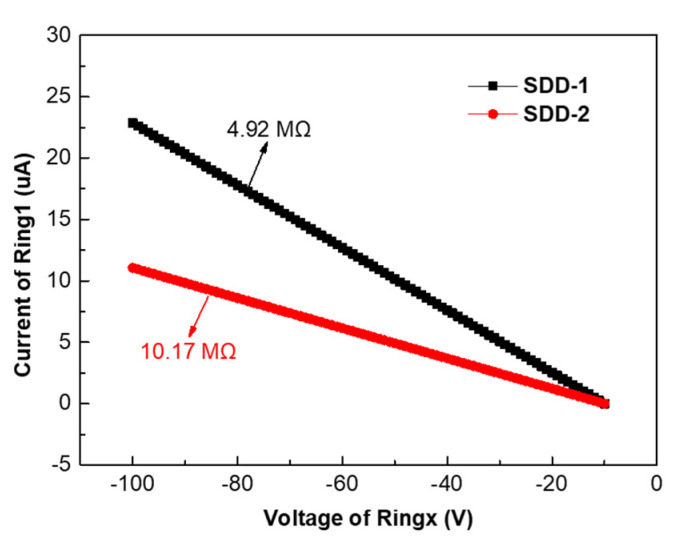
The IV characteristics of voltage dividers of SDD-1 and SDD-2.

Secondly, we studied the performance of voltage dividers of SDD-1 and SDD-2 since they play an important role in SDD. The requirements for voltage dividers are to provide a uniform drop in voltage between the drift rings and create a steady lateral drift electric field within the SDD to drive the signal electrons to the anode. Therefore, the voltage dividers need to be resistive and the IV characteristics of the voltage dividers should conform to Ohm’s law. Figure 6 shows the IV characteristics of voltage dividers of SDD-1 and SDD-2. It can be clearly seen that the IV characteristics of the voltage dividers of SDD-1 and SDD-2 obey Ohm’s law and the resistance of their voltage dividers is 4.92 MΩ and 10.17 MΩ, respectively, in the voltage range of −100 V to −10 V. It is worth noting that the resistance values of voltage dividers of SDD-1 and SDD-2 exhibit approximately a two-fold difference, which is due to their anode position being one in the center of the active region (SDD-1) and one at the edge of the active region (SDD-2), resulting in a different number of voltage dividers.

**Figure 7:**
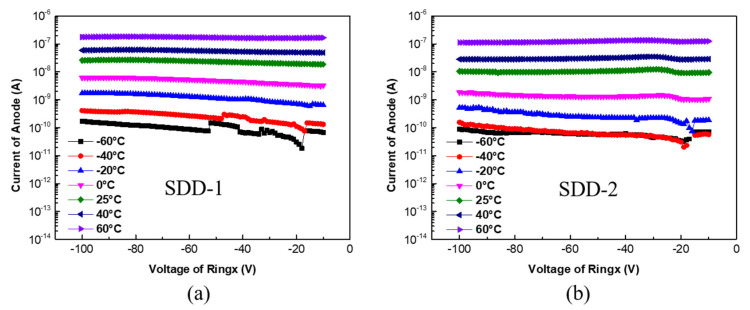
(**a**,**b**) The anode current of SDD-1 and SDD-2 at the temperature range from −60 °C to 60 °C, respectively.

Then, the anode current of SDD-1 and SDD-2 at different temperatures were tested and analyzed. As shown in Figure 7a,b, there is an obvious difference in anode current for the two devices at the temperature range from −60 °C to 60 °C. When the temperature dropped from room temperature (25 °C) to −60 °C, the anode current of SDD-1 and SDD-2 decreased by about two to three orders of magnitude. Similarly, the anode current of SDD-1 and SDD-2 increased by about two orders of magnitude as the temperature rose from room temperature (25 °C) to 60 °C. These results indicate that temperature has a strong effect on SDD anode current. In fact, SDD can be similarly equivalent to the structure in which multiple PN junctions are connected in series on both sides of the silicon substrate [28]. It is well known that the change in temperature mainly causes changes in the concentration of intrinsic carriers n_i_ in the PN junction and the leakage current I_L_ of PN junction is proportional to n_i_. Thus, the relationship between the temperature and the leakage current I_L_ of PN junction is as follows [29]:

(1) $$I_{L}\propto A_{1}n_{i}=A_{2}T^{\frac{3}{2}}\exp\left(-\frac{E_{g}}{2k_{0}T}\right)$$

where *n_i_* is the intrinsic carrier concentration, *A*_1_ and *A*_2_ are constants, k_0_ is the Boltzmann constant, *E_g_* is the bandgap of silicon. It is obvious that *I_L_* will decrease rapidly with lower temperature. Considering that SDD can be approximately equivalent to the series connection of multiple PN junctions, the reason why the anode current of SDD changes significantly with temperature can be explained. Therefore, for better device energy resolution, we can cool the SDD to a very low temperature to reduce the anode current.

**Figure 8:**
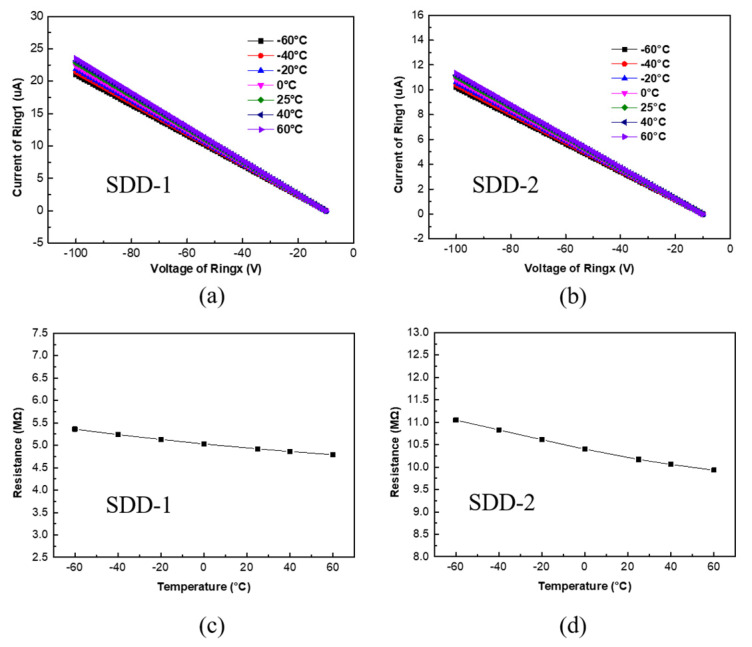
(**a**,**b**) The IV characteristics of voltage dividers of SDD-1 and SDD-2 at the temperature range from −60 °C to 60 °C, respectively. (**c**,**d**) The variation of voltage divider’s resistance of SDD-1 and SDD-2 at the temperature range from −60 °C to 60 °C, respectively.

Figure 8a,b shows the IV characteristics of voltage dividers of SDD-1 and SDD-2 at the temperature range from −60 °C to 60 °C, respectively. We can see that their I–V curves are still a straight line as the temperature increases, but the current value increases slightly. This means that the IV characteristics of voltage dividers of SDD-1 and SDD-2 also follow Ohm’s law at different temperatures, and their resistance value varies little with temperature. To further discuss the effect of temperature on the resistance value of voltage dividers, the variation of voltage divider’s resistance of SDD-1 and SDD-2 at different temperatures are depicted in Figure 8c,d. It can be clearly seen that the resistance value of voltage dividers of SDD-1 and SDD-2 decreases slightly when the temperature increases from −60°C to 60°C. The resistance value of the voltage divider of SDD-1 varies by about 0.57 MΩ compared to that of SDD-2 varies by about 1.12 MΩ. The reason why the variation of voltage divider’s resistance of SDD-2 is approximately twice as much as that of SDD-1 also can be explained by the difference in their anode positions. 

**Figure 9:**
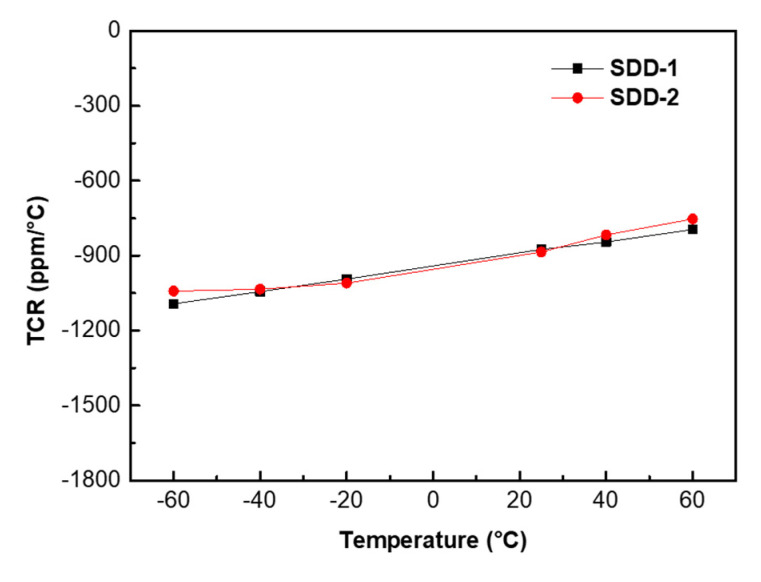
TCR values are calculated as a function of the test temperature for the voltage dividers of SDD-1, SDD-2 at the temperature range from −60 °C to 60 °C, respectively.

The working temperature of SDD usually changes with the temperature of its working environment. Thus, for SDD it is vital to have reliable operation over a wide range of temperatures. Having a temperature-stable resistor can therefore be crucial. The important qualification parameters in this respect are the resistor tolerance, expressed as the change with temperature TCR (temperature coefficient of the resistor). The TCR, which describes the variety of the voltage divider’s resistance at different temperatures, is an important parameter for the application of polysilicon films as voltage dividers of SDD. Resistors act as a voltage divider to divide the applied voltage between the inner and outer drift rings to obtain the transverse electric field in the SDD active region. It can be observed that linearity, TCR, and uniformity of the resistors have a significant influence on the generated a continuous and uniform transverse electric field to push the electrons toward the anode. Hence, if we ensure the TCR value of resistors changes little at different temperatures, the stability of the transverse electric field will be greatly improved. The TCR can be calculated by the following equation [30]:

(2) $$TCR\left(\frac{ppm}{{}^{\circ}C}\right)=\left(10^{6}\right)\frac{R-R_{0}}{R_{0}\left(T-T_{0}\right)}$$

where *TCR* is the temperature coefficient of the resistors in the ppm/°C (parts per million per degree centigrade) units, *R* and *R*_0_ are the measured resistance at temperatures *T* and *T*_0_. According to this formula, we calculated the TCR values of voltage dividers of SDD-1 and SDD-2. As shown in Figure 9, the voltage dividers of SDD-1 and SDD-2 have similar TCR values and the variety in their TCR values of them are both less than 300 ppm/◦C when the temperature range from −60 °C to 60 °C. These similar TCR characteristics of voltage dividers of SDD-1 and SDD-2 can be explained by the same voltage dividers fabrication process they adopted in our experiment. Moreover, the TCR values for the voltage dividers of SDD-1 and SDD-2 are negative, which can be explained by the variety of intrinsic carrier concentration n_i_. As is well known, the enlarged carrier concentration will decrease the resistivity of polysilicon, therefore, the resistance value will decline as temperature increases, which results in a negative TCR value. In general, the voltage dividers of SDD-1 and SDD-2 both exhibit a high stability resistance value and a low temperature coefficient at different temperatures.

**Figure 10:**
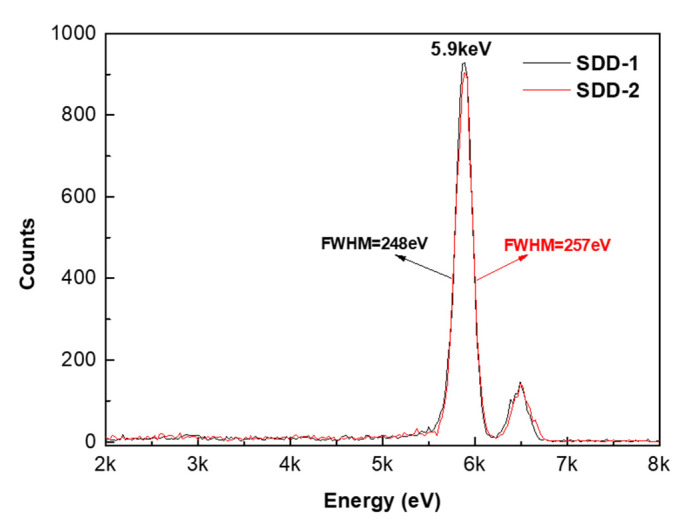
Energy spectrum of Fe-55 measured by SDD-1 and SDD-2, respectively.

Finally, the energy resolution of SDD-1 and SDD-2 was tested by placing them in a stainless-steel vacuum chamber and cooling in liquid nitrogen (see the experimental section for specific test methods). As shown in Figure 10, SDD-1 and SDD-2 have energy resolutions of 248 and 257 eV corresponding to the 5.9 keV photon peak of the Fe-55 radioactive source, respectively. This result indicated that there is little impact on the device performance irrespective of the anode positions in the square-SDD devices.

## 4. Conclusions

In summary, two different types of square-SDD with the anode located either in the center (SDD-1) or at the edge (SDD-2) of its active region were designed to explore the effect on device performance with different anode location positions. The simulation results of current density and potential distribution show that SDD-1 and SDD-2 have both formed a good electron drift path to make the anode collect electrons. We performed a comparative analysis of the device performance of the two fabricated SDDs at the temperature range from −60 °C to +60 °C, finding that the anode current of the two fabricated SDDs both decreased with the decrease of temperature, but their voltage divider characteristics exhibited high stability resistance value and low temperature coefficient, indicating that they both could provide continuous and uniform electric field at different temperatures. Finally, the energy resolution of 248 and 257 eV at 5.9 keV of the two fabricated SDDs was measured with the Fe-55 radioactive source. These results demonstrate that there is no significant impact on the device performance irrespective of the anode positions in the square-SDD devices.

## References

[1] Gatti E., Rehak P. (1984). Semiconductor drift chamber-An application of a novel charge transport scheme. Nucl. Instrum. Methods Phys. Res..

[2] Vacchi A., Castoldi A., Chinnici E.S. (1991). Performance of the UA6 large-area silicon drift chamber prototype. Nucl. Instrum. Methods Phys. Res..

[3] Castoldi A., Fiorini C., Guazzoni C., Longoni A., Strüder L. (1999). Semiconductor drift detectors: Applications and new devices. X-ray Spectrom..

[4] Lechner P., Pahlke A., Soltau H. (2004). Novel high-resolution silicon drift detectors. X-ray Spectrom..

[5] Keister J.W. (2007). Silicon photodiodes for absolute soft X-ray radiometry—Art. no. 66890U. Proc. SPIE Int. Soc. Opt. Eng..

[6] Soffitta P., Arnaud M., Murray S.S., Costa E., Muleri F., Takahashi T., Campana R., Monte E.D., Cosimo S.D., Evangelista Y. (2010). A set of X-ray polarimeters for the New Hard X-ray Imaging and Polarimetric Mission. Proc. Spie.

[7] Curceanu C., Bartalucci S., Bertolucci S., Bragadireanu M., Cargnelli M. (2011). New Experimental Limit on the Pauli Exclusion Principle Violation by Electrons—The VIP Experiment. Found. Phys..

[8] Watanabe M., Egerton R.F. (2022). Evolution in X-ray analysis from micro to atomic scales in aberration-corrected scanning transmission electron microscopes. Microscopy.

[9] Sareen R. (2021). Further measurements on light pulses using a Silicon Drift Detector R A Sareen. Using a Semiconductor X-ray Detector to Measure Light Pulses R A Sareen.

[10] Quarta G., Caricato A.P., Provenzano C., Marra M., Albanese E., Vasco G., Martino M., Maruccio L., Calcagnile L. (2022). The newly Installed IBIL (Ion Beam Induced Luminescence) Set-Up at CEDAD-University of Salento: Design and First Applications on Perovskite.

[11] Strüder L., Niculae A., Holl P., Soltau H. (2020). Development of the Silicon Drift Detector for Electron Microscopy Applications. Microsc. Today.

[12] Samber B.D., Bensellam M., Malderen S., Seiboth F., Brückner D., Garrevoet J., Falkenberg G., Jonas J.C., Vincze L. (2020). Proof-of-concept for 2D/CT element analysis of entire cryofrozen islets of Langerhans using a cryoloop synchrotron X-ray fluorescence setup. J. Anal. At. Spectrom..

[13] Lechner P., Buttler W., Fiorini C., Hartmann R., Weber U. (2000). 8-Multichannel silicon drift detectors for X-ray spectroscopy. Proc. SPIE Int. Soc. Opt. Eng..

[14] Strueder L., Hartmann R., Kemmer S., Krause N., Sampietro M. (2000). 9-Room-temperature x- and gamma-ray spectroscopy with silicon drift detectors. Proc. Spie Int. Soc. Opt. Eng..

[15] Ramsey B.D., Gaskin J.A., Elsner R.F., Chen W., Carini G.A., Geronimo G.D., Keister J., Li S., Li Z., Siddons D.P. (2012). 10-A low-power, radiation-resistant, Silicon-Drift-Detector array for extraterrestrial element mapping. J. Instrum..

[16] Gaskin J., Carini G., Chen W., Geronimo G.D., Siddons D.P. (2010). 11-The Development of a Silicon-Drift-Detector-Based X-ray Spectrometer for Remote Surface Analysis. Earth and Space 2010: Engineering, Science, Construction, and Operations in Challenging Environments.

[17] Jiang S., Tao K., Wang L., Luo W., Wang B., Song H., Li X. (2022). Fabrication of ultra-shallow junction by in situ doped amorphous silicon films and its application in silicon drift detectors. J. Phys. D: Appl. Phys..

[18] Jiang S., Jia R., Tao K., Wu Y., Liu S. (2019). High-resistance voltage dividers fabricated by thin polysilicon films in silicon drift detectors. J. Mater. Sci..

[19] Liu S., Xue Y., Jia R., Tao K., Jiang S., Wu Y., Sun H., Guo Q., Zhang L., Feng S. (2019). Design and preparation of integrated voltage divider for silicon drift detector by ion implantation. J. Mater. Sci. Mater. Electron..

[20] Wu Y., Tao K., Jiang S., Rui J., Huang Y. (2020). The investigation of surface passivation in N-type silicon and its application on Silicon Drift Detector. Chin. Phys. B.

[21] Liu W., Zhao X., Deng Z., Li F., Qi H. (2020). WASA: A low power front-end ASIC for time projection chambers in 65 nm CMOS. J. Instrum..

[22] Deng Z., He L., Liu F., Liu Y., Li Y., Yue Q. (2018). An ultra-low noise cryogenic CMOS charge sensitive preamplifier for large volume point-contact HPGe detectors. J. Instrum..

[23] He L., Hao J., Deng Z., Liu F., Liu Y., Li Y., Yue Q., Cai J. (2019). Comparison of JFET/MOS/HEMT Based Low Noise Charge Sensitive Preamplifiers for HPGe Detectors in Cryogenic Temperature. J. Phys. Conf..

[24] Wu Y.C., Jhan Y.R. (2018). 3D TCAD Simulation for CMOS Nanoeletronic Devices. 3D TCAD Simulation for CMOS Nanoeletronic Devices.

[25] Deal B., Mckenna E., Castro P. (1969). Characteristics of Fast Surface States Associated with SiO[sub 2]-Si and Si[sub 3]N[sub 4]-SiO[sub 2]-Si Structures. J. Electrochem. Soc..

[26] Wei C., Zheng L., Kraner H.W. Application of the rapid thermal process: Sintering the sputtered aluminum/silicon contact in silicon detector fabrication. Proceedings of the Nuclear Science Symposium & Medical Imaging Conference.

[27] Alok D., Baliga B. (1997). SiC device edge termination using finite area argon implantation. IEEE Trans. Electron Devices.

[28] Kovalevskii A.A., Borisenko V.E., Borisevich V.M., Dolbik A.V. (2005). Doping Effect on the Structure of Polycrystalline Silicon Films Grown via Silane Pyrolysis. Inorg. Mater..

[29] Sproul A.B., Green M.A. (1993). Intrinsic carrier concentration and minority-carrier mobility of silicon from 77 to 300 K. J. Appl. Phys..

[30] Rehak G.P. (2005). Review of semiconductor drift detectors. Nuclear Instruments and Methods in Physics Research Section A: Accelerators, Spectrometers, Detectors and Associated Equipment.

