# Correction: Luo et al. The Effects of Different Anode Positions on the Electrical Properties of Square-Silicon Drift Detector. *Micromachines* 2022, *13*, 1496

**DOI:** 10.3390/mi15020183

**Published:** 2024-01-26

**Authors:** Wei Luo, Longjie Wang, Rui Jia, Ke Tao, Bolong Wang, Xiaoping Ouyang, Xing Li

**Affiliations:** 1 Institute of Microelectronics of the Chinese Academy of Sciences, Beijing 100029, China; luowei2020@ime.ac.cn (W.L.); wanglongjie@ime.ac.cn (L.W.); taoke@ime.ac.cn (K.T.); wangbolong@ime.ac.cn (B.W.); 2 University of Chinese Academy of Sciences, Beijing 101408, China; 3 State Key Laboratory of Intense Pulsed Radiation Simulation and Effect, Northwest Institute of Nuclear Technology, Xi’an 710024, China; oyxp2003@aliyun.com

In the original publication [1], there was an error regarding the author affiliations. In addition to affiliation 1. Institute of Microelectronics of the Chinese Academy of Sciences and 3. State Key Laboratory of Intense Pulsed Radiation Simulation and Effect, Northwest Institute of Nuclear Technology, the updated affiliations should include 2. University of Chinese Academy of Sciences. The authors state that the scientific conclusions are unaffected. This correction was approved by the Academic Editor. The original publication has also been updated.
